# Gelatinous Abdomen: A Rare Case of Pseudomyxoma Peritonei Arising from Metastatic Gastric Adenocarcinoma

**DOI:** 10.7759/cureus.4666

**Published:** 2019-05-14

**Authors:** Wen Wang, Lingbin Meng, Eric Crespo, Jeffrey Adams, Manoucher Manoucheri

**Affiliations:** 1 Internal Medicine, AdventHealth Orlando, Orlando, USA; 2 Internal Medicine, AdventHealth Winter Park, Winter Park, USA; 3 Internal Medicine, Florida State University, Tallahassee, USA; 4 Internal Medicine, University of Central Florida, Orlando, USA; 5 Internal Medicine, Florida Hospital, Orlando, USA

**Keywords:** sugarbaker procedure, metastatic gastric cancer, pseudomyxoma peritonei, hyperthermic intraperitoneal chemotherapy (hipec)

## Abstract

Pseudomyxoma peritonei (PMP) is a rare condition that is typically associated with appendicular adenocarcinoma. Other origins are rarely reported. Here, we report a rare case of abdominal PMP, which originated as gastric adenocarcinoma. The prognosis of the patient consisted of pathological diagnosis, with samples from exploratory laparotomy, radiological visualization, abdominal computed tomography (CT), and abdominal magnetic resonance imaging (MRI). We employed the standard treatment protocol for our patient, which was essentially surgical, applying a cytoreductive technique, in an attempt to remove all visible evidence of the disease, plus intraperitoneal chemotherapy.

## Introduction

Pseudomyxoma peritonei (PMP) is a rare malignant growth of mucus-secreting tumor cells within the abdomen and pelvis. Typically, the primary site of PMP is the appendix. However, on rare occasions, it can metastasize from gastric and gallbladder adenocarcinomas to the peritoneal, appendix, liver, and pelvic regions, with more aggressive behavior.

## Case presentation

A 50-year-old African American male with a past medical history of gastric adenocarcinoma presented with left lower quadrant and peri-umbilical abdominal pain, which was chronic but had worsened over the two weeks prior to admission. He described it as sharp, intermittent, and non-radiating pain that was seven out of 10 in intensity. He also complained of poor appetite and vomiting at least once a day after eating for three months. He estimated that he lost 70 pounds during this same period of time. He denied fever, constipation, diarrhea, hematemesis, or blood in the stool.

He had extensive medical and surgical history. He was diagnosed with gastric adenocarcinoma by diagnostic laparoscopy, with a biopsy, in March 2012. In January 2014, he was admitted to Singapore General Hospital for extensive surgical resection. He underwent partial gastrectomy, subtotal colectomy, splenectomy, cholecystectomy, complete omentectomy, partial peritonectomy, and temporary abdominal closure. He then received another surgery during the same hospitalization, which included more extensive peritonectomy and hyperthermic intraperitoneal chemotherapy. Afterward, he received chemotherapy on and off in Canada.

On physical exam, he had a large midline incision with nodular hernias. There was mild tenderness to palpation in the periumbilical and left lower quadrant. Shifting dullness was present. The abdomen was firm, however, there was no guarding or rebound tenderness.

Initial laboratory results were significant for mild leukocytosis (WBC 11000/uL), hypokalemia (potassium of 2.7 mmol/L), acidosis (bicarbonate of 12 mmol/L), hypomagnesemia (magnesium 1.1 mg/dL ), hypoalbuminemia (albumin 1.5 g/dL), hypoproteinemia (total protein of 4.2 g/dL), and mildly elevated lipase (113 units/L). Liver enzymes and renal function test were within normal limits.

**Figure 1:**
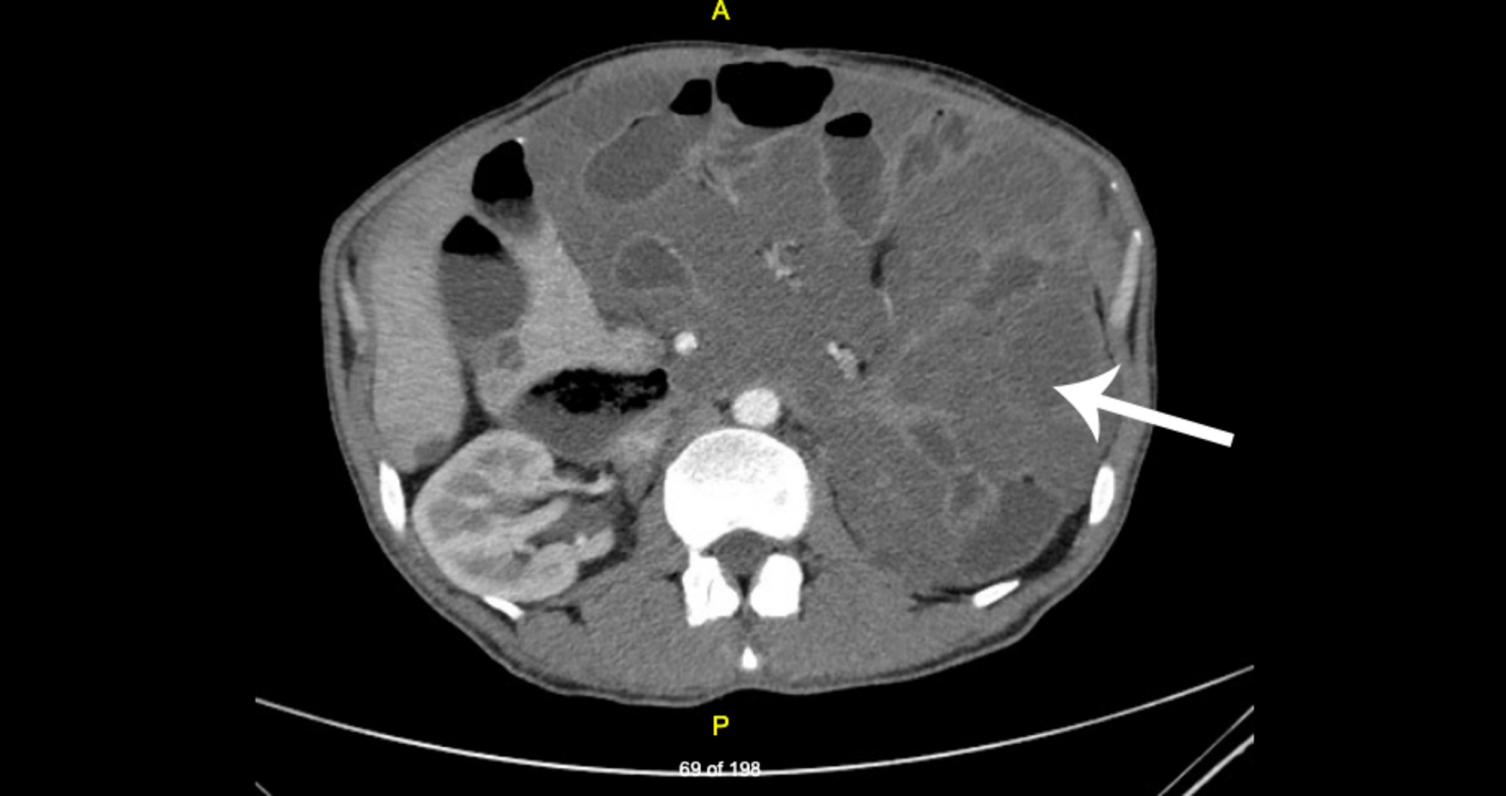
CT scan of abdomen and pelvis with contrast Diffuse pseudomyxoma peritonei in the peritoneal cavity (solid arrow) CT: computed tomography

Computed tomography (CT) scan of the abdomen and pelvis with contrast showed diffuse pseudomyxoma peritonei with a large, septated low-density mass (Figure 1) and a few calcifications along the inferomedial aspect of the left and right hepatic lobes in the region of the porta hepatis.

General surgery was consulted. The patient was deemed a potential candidate for cytoreductive surgery and hyperthermic intraperitoneal chemotherapy (HIPEC). There was one surgeon in another hospital with more experience in this procedure so the patient was transferred there for further treatment. Over the other hospital, the patient developed hemoptysis and fever. The patient was diagnosed with pneumonia and was treated with broad-spectrum antibiotics. Unfortunately, the patient was deemed not stable enough for extensive cytoreductive surgery. He was discharged to a nursing home to follow up with the surgery team outpatient. Meanwhile, the patient was offered the option of systemic chemotherapy by an oncology team as an alternative therapy in case he could not receive surgery. Subsequently, we lost follow-up of the patient, as we were unable to get in touch with him from the second month after transfer.

## Discussion

Abdominal pseudomyxoma peritonei (PMP) is, by definition, the presence of mucin produced by a tumor in the peritoneal cavity. It is considered a rare or “orphan” disease. Its occurrence is estimated to be two cases/one million/year [1].

PMP develops most often in appendix tumors and ovarian tumors; rarely is it associated with digestive system tumors. It is not clear why certain tumors tend to cause PMP while others do not. Other reported origins include the ovary, colon, stomach, pancreas, and urachus [2-3]. PMP is more often seen in patients with low-grade or intermediate-grade mucinous adenocarcinoma, unlike our rare case in which the PMP is of gastric adenocarcinoma origin. In contrast, peritoneal carcinomatosis without mucin formation is more likely to occur in high-grade adenocarcinomas [4].

About one-third of the patients with PMP have widespread involvement of the peritoneal cavity. The malignant cells have low adhesive potential and thus high propensity to spread. Dissemination is initially locoregional. The privileged sites are areas that tend to be more static. Examples are the Douglas pouch and parabolic gutters, but all abdominal organs can be involved [3].

The signs and symptoms of PMP are usually non-specific. There is no pathognomonic sign, which adds to the diagnostic difficulty. Patients can have abdominal pain or discomfort, nausea, vomiting, poor appetite, and weight loss. The complications of PMP include severe adherence between abdominal organs, small bowel obstruction, hernia, and ascites [5]. As in our case, the patient presented with diffuse abdominal pain, daily vomiting, and profound weight loss over a short period of time. He also developed a peri-umbilical hernia associated with large peritoneal tumor load.

Imaging (ultrasound, CT abdomen/pelvis) is very useful to visualize the lesions. The original tumor, disseminated implants, scalloping of the liver, peritoneal effusion, and mesenteric involvement can be seen in the images [6]. Thus we were able to utilize this imaging technique as a tool to aid in our patient's diagnosis, which revealed PMP with septation and low-density mass in the hepatic lobes.

Complete cytoreductive surgery (CCRS) with hyperthermic intraperitoneal chemotherapy (HIPEC) is the treatment of choice for pseudomyxoma peritonei (PMP) [7-8]. Pressurize intraperitoneal aerosol chemotherapy is an alternative to hyperthermic treatment, with fewer complications. Regarding our patient's case, although it was not possible to perform extensive CCRS, he could still be a candidate for chemotherapy to curtail further PMP metastasis after he recovered from his acute illness. Radiotherapy is not effective in PMP. There is no previous study to demonstrate the efficacy, tolerability, and safety of CCRS with HIPEC in gastric PMP patients. The PERISCOPE (prospective evaluation of a risk score for postoperative pulmonary complications in Europe) study is an ongoing, open-label, multicenter, dose-escalation study to investigate in this field [9]. We look forward that the patients' study outcomes would shed more light on diagnosis and strategic treatment endeavors.

The prognosis of PMP depends on its origin. With CCRS and HIPEC, for appendiceal PMP, the median survival rate is around 16.3 years while median progression-free time is about 8.2 years [10]. For gastric-origin PMP, as mentioned above, there is no data available for the survival rate in patients treated with a combination of CCRS and HIPEC. Without treatment, gastric-origin PMP has a median survival of only three to four months [9-10], which calls for an urgent need for an insight into early diagnosis and tactical treatment options.

## Conclusions

PMP is a rare condition that occurs mostly in appendicular tumors and ovarian tumors. There are reported cases of PMP from gastric and pancreatic tumors, which are even rarer sites. PMP from malignancy other than that of appendicular origin tends to be more aggressive. Complete cytoreductive surgery (CCRS) with hyperthermic intraperitoneal chemotherapy (HIPEC) is the treatment of choice.
